# *News & views:* anti-amyloid antibodies and novel emerging approaches to Alzheimer’s disease in 2023

**DOI:** 10.1186/s13024-023-00656-x

**Published:** 2023-09-25

**Authors:** Sam Gandy

**Affiliations:** 1 Department of Psychiatry and the NIA-Designated Mount Sinai Alzheimer’s Disease Research Center, Icahn School of Medicine at Mount Sinai, New York, NY 10029 USA; 2 James J Peters VA Medical Center, Bronx, NY 10468 USA; 3 Department of Neurology and Mount Sinai Center for Cognitive Health, Icahn School of Medicine at Mount Sinai, New York, NY 10029 USA

**Keywords:** Aducanumab, Bapineuzumab, Donanemab, Lecanemab

Alzheimer’s disease (AD) was described over a century ago as a disease of dementia with the presence of amyloid and tau pathologies [1], but the past year has seen the first clear evidence that effective disease-modifying anti-amyloid antibody (AAA) therapies are possible. Both aducanumab [2] (from Biogen) and lecanemab [3, 4] (from an Eisai-Biogen collaboration) earned accelerated approval from the US Food and Drug Administration (FDA) while lecanemab also earned traditional approval as well. With FDA review of donanemab [5, 6] (Lilly) expected to take place close on the heels of the first two AAA drugs, now is an apt time to consider how the drugs compare and contrast and how to talk with patients about them.

Though aducanumab was the first AAA approved (in June 2021), controversy ensued rapidly thereafter owing to questionable efficacy [2], and, though some prescriptions continue, the drug appears unlikely to earn a competitive market share, leaving lecanemab (L) and donanemab (D) as the two drugs from which practitioners will choose in the near term. While both L and D are delivered through intravenous infusions and both clear fibrillar Aβ based on amyloid positron emission tomography (PET) scan evidence, their properties diverge from there.

## Targets

The antigen targeted by L is the protofibril-forming Arctic mutant Aβ [3], while the antigen targeted by D is pyroglutamylated N-truncated Aβ(Aβ pE3) [5]. Though both these forms of Aβ aggregates are toxic, there remains no understanding of which of these conformers is more toxic, which is more relevant to the human brain in vivo, or whether the mechanism(s) of toxicity of each are similar or different. Since the two divergent Aβ targets are incompletely understood, the different antigens provide no guidance in choosing between the two drugs. It is conceivable that a combination of L and D could be superior to either drug alone. Nonetheless, both L and D are effective in clearing amyloid plaques, the main source of Aβ fibril-related toxicity.

## Clinical trial data: effect sizes and side effect profiles

In the van Dyck et al. [4] study of ~ 1800 patients, L or placebo was infused every two weeks for 18 months. The trial met its primary and five secondary endpoints, and the rate of cognitive decline was slowed by ~ 27%. With some endpoints, that rate of slowing appeared to be increasing as the 18-month trial ended, suggesting that benefit may increase over time.

The side effect profile included local injection reactions and ARIA (amyloid-related imaging abnormalities) that could be classified as either ARIA-E (for edema) or ARIA-H (for hemorrhage). The incidence of ARIA-E was 13% in subjects receiving drug vs 1.7% in those receiving placebo; for ARIA-H, those numbers were 17.3% and 9%, respectively. *APOE ε4* alleles increased the incidence of ARIA, and, in the van Dyck [4] report, the drug benefit was apparently attenuated in that patient subgroup. This is notable since the opposite was true of *APOE ε4 *carriers in the phase 2 study [7]. Three deaths from cerebral hemorrhage occurred, two during the open-label phase of the L trial, all in the context of concurrent administration of either an anticoagulant or an anti-thrombotic medication. A subcutaneous version of L is in development that may have an important impact on remote drug use and on reducing ARIA incidence. Data presented in poster form at AAIC 2023 supported this (https://media-us.eisai.com/2023-07-19-Eisai-Presents-Latest-Analysis-of-Lecanemabs-Effect-on-Biomarker-Changes-and-Subcutaneous-Dosing-at-The-Alzheimers-Association-International-Conference-AAIC-2023). Any potential option for pausing L infusion has not been assessed. The estimated cost for L alone is about $26,000, and Centers for Medicare & Medicaid Services (CMS) has recently agreed to reimburse both L and amyloid PET scans. L is currently being evaluated in the AHEAD 3–45 study to determine whether L can prevent cognitive decline in asymptomatic subjects with plasma Aβ and tau biomarker changes (https://www.aheadstudy.org/eligibility-requirements/).

In the Sims et al.study of 1736 patients [6], D or placebo was infused monthly for 76 weeks. Of the 24 assessed outcomes (primary, secondary, and exploratory), 23 were statistically significant in favor of the drug. A novel cognitive endpoint developed by the investigators (known as the iADRS for Integrated AD Rating Scale) also showed a highly significant benefit [6]. Cognitive decline was slowed by ~ 40%, and ~ 40% of subjects receiving drug showed no decline at all. An analysis of the baseline tauopathy burden revealed that subjects receiving drug were most likely to benefit if their tau burden was in a low/moderate range “sweet spot”. As with L, ARIA incidence was significantly higher in the treatment group (about 25% with drug vs 6% with placebo) and was associated with *APOE ε4* alleles [8].

## Lessons learned and next steps

Two AAAs show clear modest benefit in relatively short trials. The most important side effect is ARIA [9], and, while ARIA is considered to be manageable in the acute phase, the chronic effects of single or multiple bouts of ARIA remain to be seen. There is a concern that ARIA episodes may be associated with excess atrophy long-term, and, even if so, the potential clinical importance of excess atrophy remains to be established.

There is no obvious scientific basis for choosing between L and D at present. D might have a slightly greater reduction of cognitive decline than L in the mild cognitive impairment (MCI) group; however, its ARIA rate is also slightly higher. L reduced tau biomarkers whereas D did not. L is the first to be approved and reimbursed, but there is no reason to doubt that D is likely to achieve both of those within the next year. D has a “precision medicine” advantage in that an individual patient’s tau burden may indicate the likelihood of benefit. Practitioners and patients are likely to collaborate on choices based on availability, formulation (i.e., availability of a subcutaneous version), and frequency of dosing.

Other targets and strategies are also beginning to bear fruit. Alnylam’s antisense oligonucleotide (ASO) strategy for APP [https://www.alnylam.com/alnylam-rnai-pipeline] and Biogen’s ASO strategy for tauopathy [https://www.biospace.com/article/biogen-successfully-targets-tau-in-phase-ib-study-for-alzheimer-s/] have shown sustained protein knockdown in cerebrospinal fluid (CSF) in the ranges of 60–90%, respectively.

Triggering receptor expressed on myeloid cells 2 (TREM2) [10] is being targeted by Vigil, Denali, and Alector. This is one of the first drugs to target neuroinflammation directly. Other targets include metabolism, the neurovascular unit (e.g., Lp-PLA2 by SciNeuro Pharmaceuticals), and synaptic integrity. The eventual possibility of combining anti-amyloid, anti-tau, and other strategies remains attractive [11, 12].

## Statement of intent

These talking points were drafted as an intended service to the private patients of the author, and no endorsement(s) by the author’s employer(s) is/are implied. The views represented here are entirely the personal views of the author.

## Data Availability

N/A.

## Abbreviations

AAA: Anti-amyloid antibody
ARIA-E: Amyloid-related imaging abnormality-edema
ARIA-H: Amyloid-related imaging abnormality-hemorrhage
APOE: Apolipoprotein E
ASO: Antisense oligonucleotide
